# Muscle synergies in neuroscience and robotics: from input-space to task-space perspectives

**DOI:** 10.3389/fncom.2013.00043

**Published:** 2013-04-19

**Authors:** Cristiano Alessandro, Ioannis Delis, Francesco Nori, Stefano Panzeri, Bastien Berret

**Affiliations:** ^1^Artificial Intelligence Laboratory, Department of Informatics, University of ZurichZurich, Switzerland; ^2^RBCS, Italian Institute of TechnologyGenoa; ^3^Communication, Computer and System Sciences Department, University of GenoaGenoa, Italy; ^4^Institute of Neuroscience and Psychology, University of GlasgowGlasgow, UK; ^5^Center for Nueorscience and Cognitive Systems @UniTn, Istituto Italiano di TecnologiaRovereto (TN), Italy; ^6^UR CIAMS, EA 4532 – Motor Control and Perception Team, Université Paris-Sud 11Orsay, France

**Keywords:** muscle synergies, modularity, task-space, dimensionality reduction, motor control, robotics, review

## Abstract

In this paper we review the works related to muscle synergies that have been carried-out in neuroscience and control engineering. In particular, we refer to the hypothesis that the central nervous system (CNS) generates desired muscle contractions by combining a small number of predefined modules, called muscle synergies. We provide an overview of the methods that have been employed to test the validity of this scheme, and we show how the concept of muscle synergy has been generalized for the control of artificial agents. The comparison between these two lines of research, in particular their different goals and approaches, is instrumental to explain the computational implications of the hypothesized modular organization. Moreover, it clarifies the importance of assessing the functional role of muscle synergies: although these basic modules are defined at the level of muscle activations (input-space), they should result in the effective accomplishment of the desired task. This requirement is not always explicitly considered in experimental neuroscience, as muscle synergies are often estimated solely by analyzing recorded muscle activities. We suggest that synergy extraction methods should explicitly take into account task execution variables, thus moving from a perspective purely based on input-space to one grounded on task-space as well.

## 1. Introduction

One of the fundamental questions in motor control concerns the mechanisms that underlie muscle contractions during the execution of movements. The complexity of the musculoskeletal apparatus as well as its dynamical properties allow biological systems to perform a wide variety of motor tasks (Bizzi et al., 1992); on the other hand, such a complexity has to be mastered by efficient strategies implemented in the central nervous system (CNS). How does the CNS “choose” among the infinity of solutions of a given motor task (i.e., Bernstein problem) (Bernstein, 1967)? How are motor intentions translated into muscle activations? How can biological systems learn and plan movements so rapidly? A prominent hypothesis suggests that motor circuitries are organized in a modular fashion, so that muscle activations can be realized by flexibly combining such modules. Modularity has been observed in various forms such as kinematic strokes, spinal force fields and muscle synergies (Flash and Hochner, 2005); this paper provides an overview of the findings related to the so-called muscle synergies, as well as the application of such a concept in robotics and character animations.

Muscle synergies are defined as coordinated activations of a group of muscles^1^. It has been suggested that the CNS encodes a set of synergies, and it combines them in a task-dependent fashion in order to generate the muscle contractions that lead to the desired movement (muscle synergy hypothesis). Evidence for this organization relies on the spatio-temporal regularities observed in the EMG (Electromyography) activities of several species (Tresch et al., 2002; Bizzi et al., 2008). Since in many cases these regularities appear to be very similar across subjects and motor tasks (i.e., robustness of muscle synergies), scientists have proposed that they might reflect a modular organization of the underlying neural circuitries. Assuming that muscle activations represent the control input to the musculoskeletal system, in this context muscle synergies are implicitly defined as input-space generators (i.e., components that are able to generate the necessary input signals).

From a computational point of view, a modular organization based on muscle synergies is very attractive. The activations of many muscles is hypothetically implemented by modulating the contributions of a small set of predefined muscle synergies. Such a dimensionality reduction may simplify motor control and learning, and it may contribute to the adaptability observed in biological systems (Mussa-Ivaldi and Bizzi, 2000). This observation has recently motivated roboticists and control engineers to develop control strategies that are based on the same concept: combination of a small number of predefined actuations. In addition to the possible dimensionality reduction, the modularity of such scheme has the advantage that improved performance may be achieved incrementally by introducing additional synergies to the controller. The price to be paid is the restriction of the possible actuations to those that can be obtained by combining the synergies (i.e., synergies span set). This also implies a reduction of the possible movements that the controlled system can perform.

**Figure 1:**
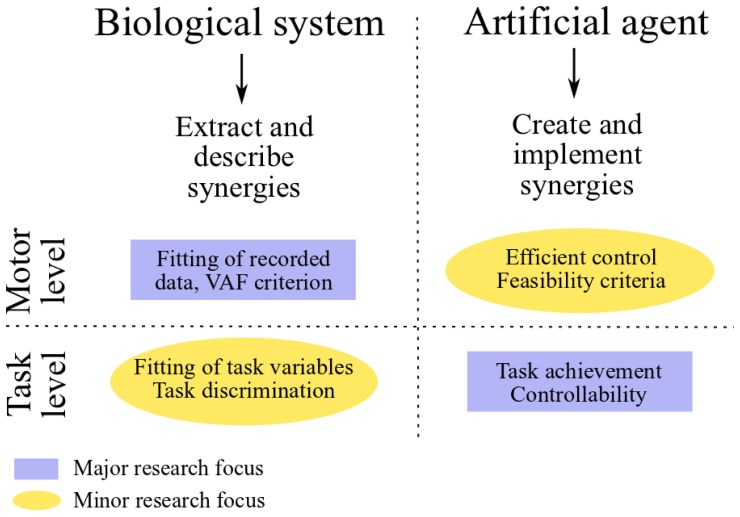
**Comparative scheme between research on muscle synergies in neuroscience and control engineering**.

In the two fields of neuroscience and control engineering, research on muscle synergies is characterized by radically different goals and approaches (see Figure 1). In the context of controlling artificial systems, the main goal is the synthesis of a small set of synergies that instantiates an effective control strategy. The obtained controller, as such, is mainly evaluated in relation to task-accomplishment, and in particular it should be able to generate a set of feasible actuations that allows the agent to perform a wide variety of tasks. In neuroscience, on the other hand, the main goal is to validate or falsify the hypothesis of muscle synergy. The typical approach consists in analyzing a dataset of recorded muscle activities, and in verifying if such a dataset is compatible with the proposed modular decomposition; the hypothetical synergies are inferred by applying a decomposition algorithm to the dataset of EMG signals. Unlike in control engineering, the major focus of this line of research resides at the motor level (i.e., the input-space of muscle activations); the evaluation of the hypothesized modular organization at the level of task is not always considered and, from our point of view, it deserves more attention. Does the set of identified muscle synergies actually lead to the task performance observed experimentally? Does it generate feasible actuations? These issues have been investigated *a-posteriori* using realistic models of the musculoskeletal systems of different species (Berniker et al., 2009; Neptune et al., 2009; McKay and Ting, 2012). Additionally, novel methodologies to deal with these challenges are starting to emerge in experimental neuroscience as well (Chvatal et al., 2011; Delis et al., 2013). We believe that a shift of paradigm from an input-space to a task-space identification of muscle synergies, which seems to be already in progress, may contribute to a better understanding of the hypothetical modularity of the CNS, and of its relationship to human learning and control. In particular in this review we argue that task-space constraints could be directly integrated in the decomposition algorithm used to extract the synergies.

This paper reviews the studies that investigate the hypothesis of muscle synergies, as well as the methods to control artificial systems that have been developed taking inspiration from this hypothesis. The organization of the paper follows the rationale developed so far. Initially, in section 2, we provide a mathematical formulation of the concept of muscle synergies, we detail different synergy models (proposed as the mechanism to generate muscle contractions), and we analyze their computational implications. In section 3 we discuss the works that evaluate the hypothesis of muscle synergies solely in the space of input-signals, and the ones that seek more direct neural evidence. Then, in section 4, we present the studies that evaluate synergies also at the task-level; this section includes robotics, characters animation, as well as neuroscience. Finally, in section 5 we offer further discussions and concluding remarks.

## 2. Models of muscle synergy

The concept of muscle synergy has been formalized in a variety of mathematical models. We will present these models in the context of controlling a generic dynamical system. This formulation is sufficiently generic to represent both the control of the musculoskeletal system and the control of an artificial agent. Furthermore, it is useful to explain the computational implications of the various synergy models, and to clarify the difference between input-space and task-space evaluation of a set of synergies.

The generic dynamical system we employ can be represented as follows:

$$\dot{x}(t)=f(x(t),t)+g(x(t),t)u(t),$$

where *t* represents time, **x**(*t*) ∈ ℝ^*n*^ is the system state variable at time *t* (e.g., angular positions and velocities of the joints), and **u**(*t*) ∈ ℝ^*m*^ is the system input at time *t* (e.g., muscle activations or joint torques). Within this framework, the variable to be controlled is denoted as **y**(*t*) ∈ ℝ^*p*^, and it is a generic function of the system state: **y**(*t*) = *h*(**x**(*t*)). The task is defined in terms of a set of constraints applied on the time evolution of this variable. Typical examples of tasks include reaching (**y**(*t*_*f*_) = **y**_*d*_ where *t*_*f*_ is the desired reaching time), and tracking (**y**(*t*) = **y**_*d*_(*t*)∀*t*, where **y**_*d*_(·) is the desired trajectory to be tracked). We refer to the task-space, as the space where the task **y**_*d*_ is defined; similarly, the input-space is the space of the input signals **u**(·). The relation between these two spaces is given by the dynamics of the system. It is now clear that a given control input should always be evaluated in relation to the error between the corresponding evolution of the controlled variable and the desired task; in other words, it should always be evaluated in task-space.

**Figure 2:**
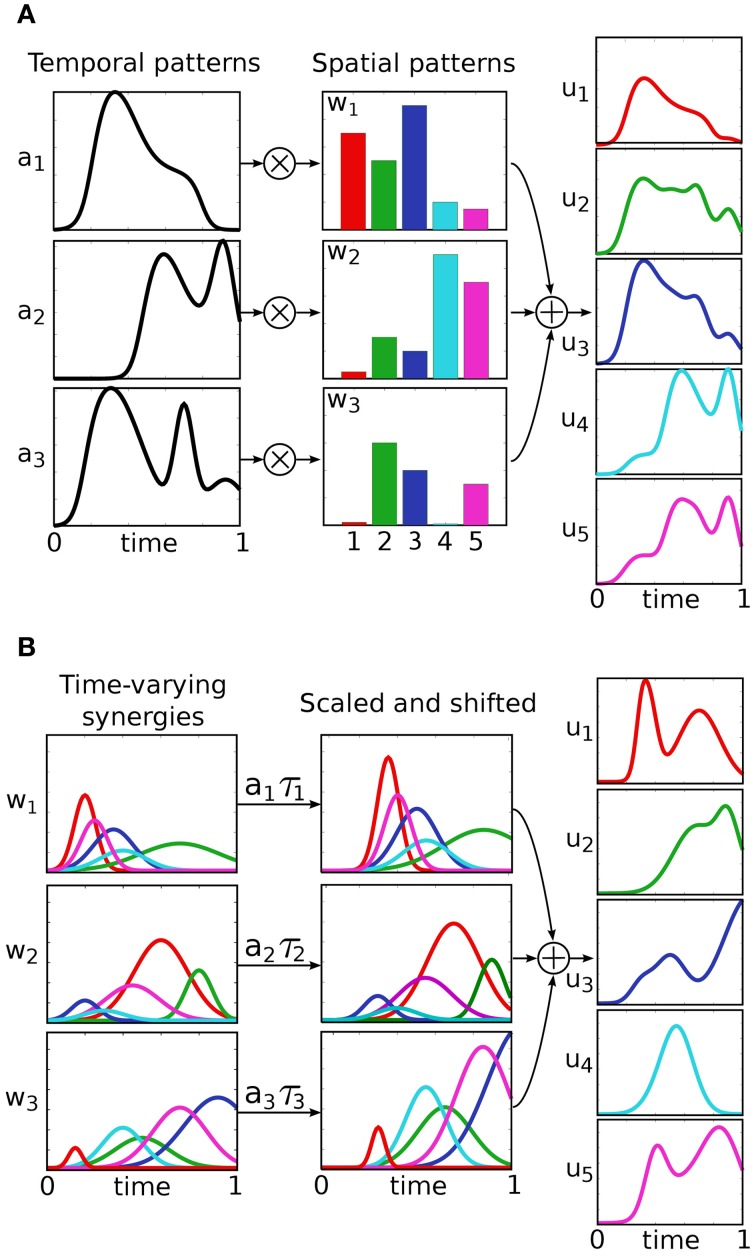
**Different models of muscle synergies.** The temporal and the synchronous models explain motor signals as linear combinations of muscle balance vectors (spatial patterns), with 1-dimensional time-varying coefficients **(A)**. In the temporal model, these coefficients serve as task-independent predefined modules, and the spatial patterns as the new (task-dependent) control input. In the synchronous model, on the other hand, the control input is represented by the temporal patterns, while the spatial patterns act as predefined modules. Finally, time-varying synergies are spatio-temporal predefined motor patterns, which can be scaled in amplitude and shifted in time by the new input coefficients **(B)**.

Classically, control inputs **u**(·) belong to the infinite dimensional space of continuous functions. Under this assumption a number of interesting control properties (e.g., controllability and observability) can be proven. The idea behind modular control, is to significantly restrict the control input-space by constraining **u**(·) to be a combination of modules, or muscle synergies. The various muscle synergy models can be distinguished based on the mathematical formalization of this combination, and they are described in the following (see Figure 2 for a schematic representation). An empirical comparison of these models is proposed by Chiovetto et al. (2013).

### 2.1. Temporal and synchronous synergies

In these models, the control input is defined as a linear combination of *k* vectors **w** ∈ ℝ^*m*^, with 1-dimensional time-varying coefficients *a*(*t*):ℝ^+^ → ℝ (Figure 2A):

(1) $$u(t)=\sum_{j\;=\;1}^{k}a_{j}(t)w_{j}.$$

Each vector **w**_*j*_ specifies a balance between the input variables (e.g., balance between muscle activations), and its coefficient *a*_*j*_(*t*) determines its temporal evolution. In the *temporal synergy model*, the coefficients {*a*_*j*_(*t*)} serve as the task-independent predefined modules, and the vectors {**w**_*j*_} represent the new (task-dependent) control input. As a result, this model reduces the control space to *k* × *m* dimensions; i.e., the *k m*-dimensional vectors **w**_*j*_ have to be appropriately specified to fulfill the desired task **y**_*d*_. Synergies are thus interpreted as the temporal patterns that are recruited selectively by different muscles. In literature, temporal synergies are also referred to as temporally fixed muscle synergies. An important special case, the *premotor drive model*, is obtained by defining the temporal coefficients as *a*_*j*_(*t*) = *A*_*j*_ ϕ(*t* − τ_*j*_). In this case, the time course of the vectors **w**_*j*_ are determined by a common function ϕ(*t*), called premotor drive or burst pulse, that can be modulated in amplitude and shifted in time. In contrast, the *synchronous synergy model* defines the task-independent synergies as the vectors **w**_*j*_. The the new control input {*a*_*j*_(*t*)} belongs to the infinite dimensional space of the one-dimensional real functions. Therefore this model, unlike the previous one, provides a dimensionality reduction only if the number of synergies is lower then the number of input variables, i.e., *k* < *m*. Synchronous synergies are co-varying group of muscles, and are also called time-invariant synergies, spatially fixed muscle synergies, or muscle modes.

### 2.2. Time-varying synergies

This model defines the control input as the superposition of *k* task-independent vector-valued functions **w**(*t*) : ℝ^+^ → ℝ^*m*^ (Figure 2B):

(2) $$u(t)=\sum_{j\;=\;1}^{k}a_{j}w_{j}(t-\tau_{j}).$$

Each synergy **w**_*j*_ can be scaled in amplitude and shifted in time by means of the coefficients *a*_*j*_, τ_*j*_ ∈ ℝ. These coefficients represent the new control input, and have to be chosen in order to accomplish the task **y**_*d*_. As a result, the new input-space is reduced to a 2 × *k* dimensional space. Neuroscientifically, these synergies are genuine spatiotemporal muscle patterns which do not make any explicit spatial and temporal separation. As such, according to this model, muscles within the same time-varying synergy do not necessarily co-vary.

## 3. Synergies as input-space generators

As discussed above, muscle synergies can be considered as input-space generators. Whether or not these generators are implemented in the CNS, and how they are eventually coordinated through the sensorimotor loops, is a main stream of research in motor neuroscience. To tackle this question, scientists have employed two main approaches. One of them is solely based on the analysis of EMG signals, therefore it can only provide indirect evidence of a modular neural organization. The other approach aims at locating the areas of the CNS where muscle synergies might be implemented, therefore providing a direct evidence. These methodologies as well as the obtained results are discussed in the following.

### 3.1. Indirect EMG-based evidence

**Figure 3:**
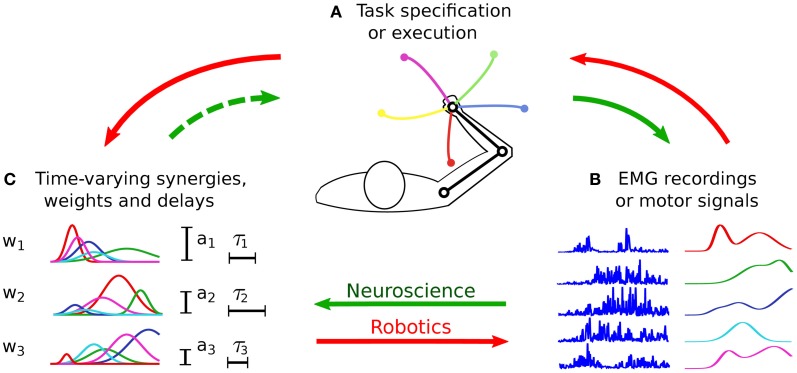
**Procedures for the identification and the testing of muscle synergies.** In experimental neuroscience (green arrows), initially a group of subjects perform the tasks prescribed by the experimenter **(A)**. The EMG signals acquired during the experiments **(B)** are then analyzed, and a dimensionality reduction algorithm is applied to obtain the synergies **(C)**. Very often such synergies are not evaluated at the task-level (dashed arrow), therefore there is no guarantee that they lead to the observed task performance. In robotics (red arrows), synergies are synthesized **(C)** based on the requirements of the desired class of tasks **(A)**. Then they are appropriately combined to generate the motor signals **(B)** to solve a specific task instance. The quality of the synthesized synergies is finally tested in terms of the obtained task performance **(A)**. Without loss of generality, the figure presents the time-varying synergy model; however, the previous description holds for all the models.

The classical approach to evaluate the hypothesis of muscle synergies consists in searching spatio-temporal regularities (i.e., synergies) in a dataset of muscle activities (Figure 3, continuous green arrows). Such a dataset is obtained by recording the EMG signals from a group of subjects/animals that are performing some prescribed motor tasks. As such, this methodology is mainly based on considerations grounded at the input level. The possibility to discriminate the various task instances from motor signals represents the only (*a-posteriori*) task-related verification of the identified synergies (see Figure 1).

Linear dimensionality reduction algorithms are employed to identify a small set of components (i.e., synergies) that approximate the EMG dataset according to the chosen synergy model (see section 2). The number of synergies to be extracted has to be specified *a-priori* by the experimenter, as it constitutes an input parameter of the decomposition algorithm. The choice of the decomposition algorithm to be used depends on the assumptions made on the nature of the hypothetical muscle synergies (e.g., non-negativity, orthogonality, statistical independence etc.) (Ting and Chvatal, 2010). Principal component analysis (PCA) (Mardia et al., 1980) looks for orthogonal synergies that account for as much of the variability in the data as possible. Similarly, factor analysis (FA) (Darlington, 1968) seeks the smallest set of synergies that can account for the common variance (correlation) of a set of muscles. Independent component analysis (ICA) (Bell and Sejnowski, 1995) maximizes the statistical independence of the extracted components, thus it assumes that synergies represents independent information sources. Non-negative matrix factorization (NMF) (Lee and Seung, 1999) enforces the extracted synergies and their activation coefficients to be non-negative; this constraint reflects the non-negativity of neural and muscle activations (“pull-only” behavior). Additionally, NMF does not assume that the generators are statistically independent, thus it is more compatible with the observation that activations of multiple synergies are correlated (Saltiel et al., 2001). Finally, the extraction of time-varying synergies is performed by an NMF-based algorithm developed *ad-hoc* that allows the components to be shifted in time (d'Avella and Tresch, 2002).

To assess the quality of the extracted synergies, the so-called VAF (Variance Accounted For) metric is typically used (see Figure 1). VAF quantifies the percentage of variability in the EMG dataset that is accounted for by the extracted synergies. High values of VAF indicate good reconstruction of the recorded EMGs, which lends credit to the extracted synergy set; low VAF values cast doubt on the extracted synergies, indicating that they do not explain a large part of the EMG variance. This metric is also used for determining the dimensionality of the synergy space. The criteria used for this purpose rely on the assumption that most of the EMG variability is attributable to task-dependent muscle activations, whereas a small portion is due to several sources of noise. Under this assumption, the number of synergies is defined either by the point where the VAF-graph (i.e., the curve that describes the trend of the VAF as function of the number of synergies, which increases monotonically) reaches a threshold level (e.g., 90%) (Torres-Oviedo et al., 2006), or by its flattening point, i.e., the point where a drastic decrease of slope is observed. Such an “elbow” is in fact interpreted as the point that separates “structured” and noise-dependent variability, and therefore it can be used to define the minimum number of synergies that capture the task-related features (d'Avella et al., 2006; Tresch et al., 2006). Besides the VAF metric, other metrics [e.g., log-likelihood (Tresch et al., 2006)] have been proposed to evaluate the effectiveness of extracted synergies (still in input-space); a thorough discussion of these metrics is beyond the scope of the present review. As depicted in Figure 1, this indirect methodology is mainly restricted to the analysis of input-level data. A complementary metric based on single-trial task-decoding techniques has been proposed by Delis et al. (2013).

A significant amount of experiments has been conducted in frogs, cats, primates as well as humans in order to test the validity of the above-mentioned synergy models, and by extension, of the muscle synergy hypothesis itself. A pioneering study showed that a small set of synchronous muscle synergies could generate a large number of reflexive motor patterns produced by cutaneous stimulations of the frog hindlimb (Tresch et al., 1999). This study also demonstrated that microstimulations of the spinal cord produced very similar muscle synergies to the ones generated by the freely moving animal. Qualitatively similar synergies were also found by intraspinal microstimulation (Saltiel et al., 2001). The above analysis was then extended in order to identify spatiotemporal patterns of muscle activities (i.e., time-varying muscle synergies) (d'Avella et al., 2003). A few time-varying synergies were shown to underlie the muscle patterns required to let the frog kick in different directions, and their recruitment was directly related to movement kinematics. These findings were further generalized to a wide variety of frog natural motor behaviors such as jumping, swimming, and walking; evidence for both synchronous and time-varying synergies was reported (d'Avella and Bizzi, 2005). Additionally, this study revealed that some synergies are shared across motor behaviors, while others are behavior specific.

The synergy models described in section 2 do not include sensory feedback, however, the original experiments on animals involved sensory-triggered reflexive movements. In fact, only a few studies have systematically investigated the influence of sensory feedback in the muscle synergy organization. Cheung et al. (2005) analyzed the EMG signals collected from the bullfrog during locomotor behaviors before and after having interrupted its sensory pathways (i.e., deafferentation). Their findings support the existence of centrally organized synchronous muscle synergies that are modulated by sensory inflow. Further support was provided by showing that an appropriate modulation of the synergy activations could explain immediate motor adjustments, and that these synergies were robust across different dynamic conditions (Cheung et al., 2009a). A discussion on the role of sensory feedback is provided in section 5.

A number of studies have examined the generalization of the above results to other species. In primates, Overduin et al. (2008) found that three time-varying synergies described a large repertoire of grasping tasks. Shape and size of the grasped objects were shown to modulate the recruitment strength as well as the timing of each synergy. In this way, this study validated that time-varying synergies account for salient task differences, and their activations can be tuned to adapt to novel behavioral contexts. Along the same lines, Brochier et al. (2004) provided further support for such a robust and distinctive synergistic organization of primates' muscle patterns during grasping. Analysis of single-trial EMG signals demonstrated that the time-varying activation of three synchronous synergies was reproducible across repetitions of the same grasping task and allowed unequivocal identification of the object grasped in each single trial. In cats, Ting's group showed that muscle synergies could be mapped onto the control of task-level variables; such experiments will be detailed in section 4.2.

The framework of muscle synergies has been successful also in characterizing the spatio-temporal organization of muscle contractions during human reaching tasks. Muscle patterns observed during movements in different directions (d'Avella et al., 2006) and speed (d'Avella et al., 2008) were accurately reconstructed by appropriate linear combinations of synergies, which appeared very similar across subjects. The synergies that were extracted from muscle activities during unloaded reaching (i.e., subjects did not hold any load in their hands) accounted for the EMG signals obtained during loaded conditions. The recruitment of the individual synergies, as well as their onset time, were consistently modulated with movement direction, and did not change substantially with movement speed. This observation was further confirmed by Muceli et al. (2010); in this study a small set of specialized synchronous synergies was able to explain a large set of multijoint movements in various directions. Finally, visually guided online corrections during center-out reaching were tested recently. The synergistic strategy was shown to be robust and more effective in explaining the corrective muscle patterns than the individual muscle activities (d'Avella et al., 2011). Furthermore, it was shown that to correct ongoing reaching movements, the CNS may either modulate existing synergies (d'Avella et al., 2011), or reprogram new ones (Fautrelle et al., 2010).

Roh et al. (2012) showed that an appropriate set of synergies could reconstruct the average patterns of muscle activation observed during isometric forces production in humans. The EMG signals were obtained for different force magnitude, directions and initial postures. The extracted synergies were very similar across conditions, and they were able to explain the corresponding datasets. Each synergy seemed to underly a specific force direction, while its activation coefficient appeared correlated to the force magnitude. In another series of experiments, a small set of synchronous synergies was able to explain static hand postures and discriminate the shapes of grasped objects (Weiss and Flanders, 2004). Moreover, a few time-varying synergies succeeded in revealing the spatiotemporal patterns of muscle activity during hand shape transitions, as in fingerspelling (Klein Breteler et al., 2007).

A relevant series of experiments showed that muscle activations involved in human postural control can be explained in terms of combinations of muscle synergies. A set of synchronous muscle synergies was able to explain muscle activations involved in postural stabilization; the EMG variation observed among trials and perturbation directions was accounted for by appropriate modulations of the synergies activation coefficients (Torres-Oviedo and Ting, 2007). In order to verify that the extracted synergies did not depend only on the specific biomechanical context, in a new experiment a set of subjects were asked to react to support perturbation from different postural configurations (Torres-Oviedo and Ting, 2010). The extracted synergies were very similar across the different conditions; however, in some cases task-specific muscle synergies needed to be added to the original synergy set to obtain a satisfactory EMG reconstruction. As the various postures lead to different patterns of sensory inflow, these results rule out the possibility that the observed synergies are only determined by specific patterns of sensory stimulations. On the contrary, they support the hypothesis that different muscle postural responses are generated by task-related modulations of the synergy activation levels. Such a hypothesis found evidence in the experiments performed by Safavynia and Ting (2012), where the temporal recruitment of the identified synchronous muscle synergies were explained by a mathematical model that explicitly takes into account the kinematic of the subject's center-of-mass (CoM). The authors then concluded that synchronous muscle synergies are recruited according to an estimate of task-related variables. The same model was previously used to fit the activations of each muscle independently during the same postural perturbation tasks (Welch and Ting, 2007). Related to postural control, Krishnamoorthy and colleagues analyzed the muscle activations that underly shifts of the centers of pressure (COP) of standing subjects (Krishnamoorthy et al., 2003a,b). In this experiment three “muscle modes,” extracted by means of PCA, explained most of the variability of the integrated EMG signals. Such components are equivalent to synchronous muscle synergies as defined in section 2, and they are characterized by the authors as the independent elemental variables that are controlled synergistically (in the sense of the UMH) by the CNS to stabilize the COP. Specifically, the model assumes that the location of the COP is modified by linear combinations of the M-modes, and their mixing coefficients represent the independent variables controlled by the CNS. Perreault et al. (2008) examined the organization of reflexes involved in postural stabilization in both stiff and compliant environments; although reflexive responses are modulated by the direction of perturbation, they showed that the synchronous muscle synergies appear very similar across conditions.

Another scenario that provides evidence to the hypothesis of muscle synergies is human locomotion (Ivanenko et al., 2006a; Lacquaniti et al., 2012b). Ivanenko et al. (2004) showed that five temporal synergies could reconstruct the muscle activity involved in locomotion tasks. These patterns are robust across walking speeds and gravitational loads, and they relate to foot kinematics (Ivanenko et al., 2003). Additionally, the same temporal synergies (accompanied by additional ones) were observed during the coordination of locomotion with additional voluntary movements (Ivanenko et al., 2005). Similar results have been reported in other locomotor behaviors such as running (Cappellini et al., 2006) and pedaling (Hug et al., 2011).

Finally, some experiments have investigated how the hypothetical synergy organization of the CNS evolves during onthogenetic development (Lacquaniti et al., 2012a). Dominici et al. (2011) observed that the two temporal synergies identified in stepping neonates are retained through development, and they are augmented by two new patterns first revealed in toddlers. The final set of synergies was observed in several animal species, consistent with the hypothesis that, despite substantial phylogenetic distances and morphological differences, locomotion is built starting from common temporal synergies. This conclusion was also supported by the comparison of temporal synergies extracted from young and elderly people, which revealed no significant effect of aging on synergy compositionality and activation (Monaco et al., 2010).

### 3.2. Direct neural evidence

The studies presented so far support the existence of synergistic muscle activations during the sensorimotor control of movements. However, these methods are indirect, in the sense that the presence of synergistic structures within the CNS can only be inferred. What remains to be tested is whether the uncovered muscle organization is neurally implemented in the CNS and, if so, in which areas. Alternatively, one could argue that the extracted synergies represent a phenomenological output of the motor coordination required for movement execution. For instance, recently Kutch and Valero-Cuevas (2012) designed carefully thought experiments and simulations to show that muscle synergies can be observed even if the nervous system does not control muscles in groups. The authors demonstrated that muscle synergies, as detected via dimensionality reduction methods (see section 3.1), may originate from biomechanical couplings and/or from constraints of the task. Similar conclusions were already reached by Valero-Cuevas et al. (2009), who showed that the observed within-trial variability of EMG data underlying the production of fingertip forces, was incompatible with the (unique) associated muscle synergy that would have been extracted. Although these findings do not directly falsify the muscle synergy hypothesis, they cast at least some doubts about the sole neural origin of modularity.

This underlines the need for a more critical assessment of the validity of the muscle synergy hypothesis. In this direction, a number of recent studies sought evidence for a neural implementation of muscle synergies, and examined which regions of the CNS may express synergies and their activations. This question has been addressed by attempting to relate neural activity with simultaneously recorded muscle activity during performance of different motor tasks. Using such an approach, Holdefer and Miller (2002) provided direct support for the existence of neural substrates of muscle synergies in monkey's primary motor cortex. In particular, they studied the activity of neurons and muscles during the execution of a variety of reaching and pointing movements, and they found that the discharge of individual neurons represents the activation of functional groups of muscles. In addition, Hart and Giszter (2010) showed that some interneurons of the frog spinal cord were better correlated with temporal synergies than with individual muscles. Therefore, they suggested that these neural populations constitute a neural basis for synergistic muscle activations (Delis et al., 2010). Another study demonstrated that the sequential activation of populations of neurons in the cat's motor cortex initiates and sequentially modifies the activity of a small number of functionally distinct groups of synergistic muscles (Yakovenko et al., 2010). Similarly, Overduin et al. (2012) showed that microstimulations of specific regions of the motor cortex of two rhesus macaques corresponded to well-defined spatial patterns of muscle activations. These synchronous synergies were very similar to those extracted from the same animals during natural reaching and grasping behaviors. Extending this research line in the context of motor learning, Kargo and Nitz (2003) showed that early skill learning is expressed through selection and tuning of primary motor cortex firing rates, which specify temporal patterns of synergistic muscle contractions in the frog's limb. Finally, Roh et al. (2011) analyzed the muscle patterns of the frog before and after transection at different levels of the neuraxis: brain stem, medulla and spinal cord, respectively. They found that medulla and spinal cord are sufficient for the expression of most (but not all) muscle synergies, which are likely activated by descending commands from supraspinal areas. Similarly, Hart and Giszter (2004) examined the compositionality of temporal synergies in decerebrated and spinalized frogs. Their results indicated that in both cases temporal synergies consisted of pulsed or burst-like activations of groups of muscles. They also showed that brainstem frogs had more focused muscle groups and showed richer behaviors than spinalized equivalents.

In humans, the main approach to locate hypothetical muscle synergies has been to analyze brain-damaged patients. Comparing the synergies extracted from healthy and brain-damaged subjects could provide hints about the neural centers involved in the synergistic control of muscles. In this vein, examining motor tasks involving arm and hand movements, Cheung et al. (2009b) showed that the synchronous synergies extracted from the arm affected by a stroke were strikingly similar to the ones extracted from the unaffected arm, concluding that muscle synergies were located in regions of the CNS that were not damaged. In a second study involving subjects with more severe motor impairment (Cheung et al., 2012), they found that synchronous synergies may be modified according to three distinct patterns—including preservation, merging, and fractionation of muscle synergies—reflecting the multiple neural responses that occur after cortical damage. These patterns varied as a function of both the severity of functional impairment and the temporal distance from stroke onset. Similarly, Roh et al. (2013) found systematic alterations of the upper limb synergies involved in isometric force production in stroke patients with severe motor impairment. However, these alterations did not involve merging or fractionation of normal synergies. Clark et al. (2010) investigated the modular organization of locomotion in stroke patients. They found a coordination pattern consisting of fewer synchronous synergies than for the healthy subjects. These synergies resulted from merging of the synergies observed in healthy subjects, suggesting reduced independence of neural control signals. In contrast, Gizzi et al. (2011) demonstrated that the temporal waveforms of the synergy activation signals, but not the synchronous synergies, were preserved after stroke.

Finally, a different but worth-mentioning approach has been the attempt to map the activity of leg muscles onto the alpha-motoneuron pools along the rostrocaudal axis of the spinal cord during human locomotion (Ivanenko et al., 2006b, 2008). Using this procedure, the authors could infer the temporal and spatial spinal motor output for all the muscles of the legs during a variety of human walking conditions, and relate them to the control of task-relevant variables such as center of mass displacements. Overall, their findings support the existence of some spinal circuitry that implement temporal synergies. The strength of this approach resides in the explicit use of anatomical and clinical charts that document the innervation of the lower limb muscles from the lumbosacral enlargement (Cappellini et al., 2010).

## 4. Synergies from the perspective of the task-space

### 4.1. From input-space to task-space: general rationale

The methodology presented in section 3.1 undeniably led to many crucial insights, however, it does not guarantee that the extracted synergies account for the observed task performance. VAF-like metrics only measure the capability of the synergies to reconstruct/fit the dataset of recorded “input-signals” (i.e., EMG data). Moreover, in some studies, such signals are averaged across movement repetitions. In this case, the VAF constitutes an average indicator, and it does not quantify the capability of the synergies to reconstruct each individual trial (Ranganathan and Krishnan, 2012). Since the musculoskeletal apparatus is a non-linear system, these approximations of the recorded muscle activities may not lead to the observed task performance (Broer and Takens, 2011; section 1.1), a condition that would harm the validity of the hypothesized modular control structure. On a similar note, the extracted synergies might generate unfeasible joint torques. Finally, even if the dataset of muscle activity is very well approximated, additional muscles that are not recorded during the experiment might have a crucial role in the generation of the movement. These issues emerge because the dynamics of the musculoskeletal system (i.e., its input–output relation) is not directly taken into account in the synergy decomposition algorithms.

In this section we review those works that attempt to relate muscle synergies to performance variables defined in task-space. Initially, we present the concepts of functional synergies and spinal force fields. The former constitutes a valid strategy to include the task variables into the classical EMG-based analysis; the latter provides task-based evidence for neurally implemented muscle synergies. Then, we discuss some studies that, in the context of biomechanics, employ plausible musculoskeletal models to test the movements obtained from experimentally extracted muscle synergies. Finally, we shift our attention to robotics and characters animation. In these fields, the main challenge is the synthesis of a small set of synergies that reduces the dimensionality of control and, at the same time, spans a subspace of actuations that allows the agent to perform a wide variety of tasks (Figure 3, red arrows). Ideally, the synthesized synergies should preserve controllability and reachability of the system. Loosely speaking, this means that any desired system state can be reached by an appropriate control input (i.e., combination of synergies) in a finite amount of time. At the motor level, it is important that the synergies generate feasible actuations; additional properties, such as the generation of optimal control signals, may also be desirable (see Figure 1).

### 4.2. Functional muscle synergies and spinal force fields

In most of the works presented so far, the functional role of muscle synergies is estimated *a-posteriori* by analyzing the dependence of the recruitment coefficients (i.e., gain and/or onset time) on the task conditions (e.g., reaching direction, force magnitude and direction, perturbation direction). Typically, each muscle synergy is assumed to underlie the task-level functionality observed in conjunction with the higher values of its activation coefficient. As an example, the analysis of directional tuning curves illustrated that some of the synergies were directly related to reaching in specific directions (d'Avella et al., 2008). A different approach is taken by a pool of studies which define the concept of functional synergies; i.e., components, typically extracted by means of NMF, of a dataset containing both EMG signals and measurements of defined task-related variables. As a result, each component consists of two elements: a balance of muscle contractions (i.e., synchronous muscle synergy), and the evolution of the task-related variables induced by such a muscle synergy (task-related vector). In our view, the concept of functional synergies provides a way to tackle the drawbacks of input-based extraction algorithms: if a set of functional muscle synergies extracted from a training-set is able to reconstruct both the EMG and, more importantly, the task-related signals observed in another set of data (testing set), then it is more likely that combinations of such muscle synergies will generate the appropriate control signals to perform the task successfully.

Functional muscle synergies were analyzed in the context of postural tasks in experiments with humans (Chvatal et al., 2011) and cats (Ting and Macpherson, 2005; Torres-Oviedo et al., 2006). The task-related variables were defined as the forces measured under the feet of the subject, which reacted to unexpected motions of the support surface. The experiments showed that each subject exhibited the same functional synergies for both stepping and non-stepping responses to perturbations (Chvatal et al., 2011), suggesting that a common pool of muscle synergies, with specific biomechanical functionalities, can be used by the CNS to drive the motion of the CoM independently of the subject's behavioral response. The functional synergies extracted from the non-stepping data were able to reconstruct the EMG signals, the CoM acceleration and the direction (not the magnitude) of the forces recorded during stepping responses; however, an additional stepping-specific muscle synergy was needed to increase the quality of EMG reconstruction. Generality and robustness of functional synergies were also analyzed in postural experiments with cats (Torres-Oviedo et al., 2006). In this study, a group of cats experienced both translations and rotations of the support surface. Functional muscle synergies were extracted from a dataset containing EMG signals and ground forces observed for different postural configurations (i.e., distances between the anterior and the posterior legs). The functional synergies extracted during surface translations for the most natural posture were able to reconstruct the data observed in all the other conditions (i.e., different postural configurations and surface rotations). Moreover, functional synergies appeared very similar across subjects. These results suggested that each muscle synergies implements a specific biomechanical functionality (Ting and Macpherson, 2005), which is general across tasks and robust across subjects.

The methodology proposed by Ting and colleagues is undoubtedly a valuable attempt to identify muscle synergies that are directly related to task execution, however, it presents some limitations. First, NMF extracts non-negative components and coefficients; while this constraint is well justified at the muscle activation level (see section 3.1), task variables may exhibit negative values. Second and more important, in addition to a linear superposition also at the task-level, this decomposition procedure assumes that both EMG signals and task-variables are generated with the same mixing coefficients. Although it is possible to obtain a good fit of a given dataset, due to the non-linearity of the musculoskeletal system, this assumption does not hold in general.

A radically different approach to investigate the modularity of motor circuitries consists in analyzing the so called spinal force fields. This method is grounded on the seminal discovery that electrical stimulations of individual regions of the frog's spinal cord produce peculiar isometric endpoint forces that depend on the posture of the limb; the direction of the force vectors within each of these fields is invariant over time, while their magnitudes are characterized by a specific time evolution. Additionally, each of these force fields features a specific point of convergence. Structures with these characteristics can be generated by groups of coactive and linearly covarying muscles (Giszter et al., 1993; Mussa-Ivaldi et al., 1994). In particular, only a small subset of all the possible muscle combinations leads to robust and convergent force fields (Loeb et al., 2000). Therefore, the observation of such characteristics in an experimentally measured force field can be regarded as an indirect evidence for spinally implemented temporal muscle synergies (see section 2). Kargo and Giszter (2000b) showed that rapid corrections of movements in wiping frogs can be explained as linear combinations of spinal force fields. Additional evidence was obtained by examining the force fields generated by frogs (Giszter and Kargo, 2000) and turtles (Stein, 2008) that exhibited deletion of motor patterns. Another method to investigate the nature of spinal circuits is the analysis of feedback mechanisms in relation to force fields. Different external excitations of the frog's muscle spindles during wiping reflexes led to structurally invariant force fields across time. Furthermore, the bursts of muscle activity underlying the wiping behavior and the balance of activations across muscles were not altered by the spindle feedback. Instead, feedback regulated the amplitude and the timing of each single burst. Since these variables did not covary across the pulses, the authors concluded that individual premotor drive pulses and not time-varying synergies are the units of spinal activity (Kargo and Giszter, 2008). Such hypothetical neural organization is compatible with the synergy scheme proposed by Drew et al. (2008) and Krouchev et al. (2006) for locomotive behaviors. These schemes allow a sequential activation of coordinated groups of muscles, a mechanism that can be implemented in the premotor drive model by modulating the onset time of the bursts. Spinal force fields are effectively task-level representations of hypothetical neural modules, however, this methodology does not provide any estimate of what the corresponding muscle synergies may look like. Moreover, the relation between linear combinations of muscle synergies and linear combinations of force fields is far from being trivial.

### 4.3. Neuromechanical modeling

Although many studies in experimental motor control provide support to the hypothesis of muscle synergies, it is hard to test whether the proposed control model can effectively lead to the task performance observed experimentally and generalize to other tasks. This issue can be tackled computationally by employing biologically plausible models of the musculoskeletal apparatus.

A pool of studies investigate if a modular organization like the synchronous synergy model can explain a complex task like human walking (Neptune et al., 2009; McGowan et al., 2010; Allen and Neptune, 2012). A set of synergies are identified from a dataset of recorded EMG signals by means of NMF. Such “modules” are then used to generate the muscle control inputs to a musculoskeletal model of the human legs. Using these synergies as a first guess, a numerical procedure optimizes the relative level of muscle activation within each module and the time course of the weighting coefficients; the objective is to minimize the difference between the results of the forward simulation and the values of the task variables measured experimentally. The walking kinematic and the ground reaction forces are well reproduced by 5 modules, if the motion is constrained in 2D (Neptune et al., 2009), and 6 modules for 3D walking (Allen and Neptune, 2012). Additional simulations reveal that the muscle groups identified during normal walking are able to emulate walking tasks with very different mechanical demands (i.e., change in mass and weight of the models) (McGowan et al., 2010). These results agree with the theoretical considerations formulated by Nori et al. (2008). Finally, this research shows that each module is associated to a specific biomechanical functionality (e.g., body support, forward propulsion, leg swing and balancing).

Related results are presented by McKay and Ting (2008, 2012). The goal of these studies is to predict the patterns of muscle activities and the ground reaction forces observed experimentally in unrestrained balance tasks with cats (Torres-Oviedo et al., 2006). Muscle contractions for an anatomically-realistic musculoskeletal model of the cat are computed; the used optimization procedure constrains task-related variables (i.e., center of mass) to the experimental results. Although many different cost functions are tested, the best predictions are achieved by minimizing control effort (i.e., total squared muscle activation). Predictions improve if muscle contractions are constrained to linear combinations of the experimentally derived synergies (Torres-Oviedo et al., 2006); however, the overall control effort increases, and the range of admissible ground forces reduces substantially. Furthermore, these studies validate the assumption made by Torres-Oviedo et al. (2006) that the ground reaction forces associated to each synergy rotate as a function of the limb axis. These results suggest that muscle synergies are feasible physiological mechanisms for the implementation of near-optimal or “good-enough” motor behaviors (de Rugy et al., 2012).

Kargo et al. (2010) employed a biomechanical model of the frog hindlimb to test whether the model of premotor drive could account for the wiping behavior observed experimentally (Kargo and Giszter, 2008). The parameters of the premotor drive model (i.e., muscle groups, pulse time course, and amplitude and phasing of the single synergies) are initially identified to reproduce experimental isometric forces and free limb movement kinematics. As expected, starting from different limb postures the derived feedforward control fail in driving the simulated limb toward the target. However, as showed by Kargo and Giszter (2008), appropriate feedback modulations of the amplitude and the phase shift of the drive burst, and the adjustment of muscle balance based on the initial configuration of the limb, are enough to generate successful muscle activations. Furthermore, the limb trajectories obtained with and without feedback are very similar to those observed in intact and deafferented (Kargo and Giszter, 2000a) frogs, respectively. These results support the model of premotor drives, in which feedback mechanisms preserve the duration of the pulses.

Berniker (2005) analyzed mathematically the control scheme of muscle synergies and proposed a principle for its formation (Berniker et al., 2009). A linear reduced-dimensional dynamical model that preserves (to the best extent possible) the natural dynamic of the original system is initially computed. Synergies are defined as the minimal set of input vectors that influence the output of the reduced-order model (Berniker, 2005), and that minimally restrict the commands (and the resulting responses) useful to solve the desired tasks (Berniker et al., 2009). Practically, this set is found by optimizing the synergy matrix over a representative dataset of desired sensory-motor signals. This method was able to synthesize a set of synergies for the model of the frog hindlimb that were very similar to the ones observed experimentally (Cheung et al., 2005). Furthermore, the synergy-based controller produced muscle activations and kinematic trajectories that were comparable with the ones obtained with the best-case controller (that can activate each muscle independently).

### 4.4. Robotics and character animation

In the context of robotics and characters animation, the concept of muscle synergies is appealing as it provides a strategy to reduce the number of variables to be controlled (synchronous synergy model), or more generically, the dimensionality of the control signals (time-varying synergy model). Animated characters are embedded in physical environments (i.e., dominated by physics laws) thus the associated control problem is totally equivalent to the control of a musculoskeletal model or of a humanoid robot. In this section we present the works that have been carried out in these fields of research.

The work proposed by Mussa-Ivaldi (1997) is one of the first attempts to develop a controller based on the modularity observed in biological systems (Mussa-Ivaldi and Giszter, 1992). The idea is that the motion of a kinematic chain can be determined by a force field applied to its end effector. Inspired by the experiments performed by Giszter et al. (1993), such a force-field results from the linear combination of basic fields, each characterized by a single equilibrium point in operational space. Results show that, for a simulated planar kinematic chain, an appropriate choice of the basis-field coefficients can produce a wide variety of end-effector trajectories. Similarly, Matarić et al. (1999) used force fields to drive joint torque controllers on a rigid-body animated character (Matarić et al., 1998a,b).

Although the concept of spinal-force field is very similar, Mussa-Ivaldi's work does not directly use the notion of synergy as defined in section 2. A step forward is taken by Nori and Frezza, who propose a mathematical formulation for a set of actuations (i.e., synergies) that comply with the hypothetical properties of spinal-force fields (Mussa-Ivaldi and Bizzi, 2000). The mathematical description of the synergies is derived from the closed-form solution of an optimal control problem. Additionally, a feedback controller assures that the system follows the desired trajectory toward the synergy equilibrium position. It is proved that the proposed formulation guarantees system controllability^2^. The synthesized synergies are successfully tested on a simulated two-degrees-of-freedom (dof) planar kinematic chain (Nori, 2005; Nori and Frezza, 2005).

The idea that each synergy solves a well-defined control problem [e.g., to lead the system to a specific equilibrium position (Nori and Frezza, 2005)], appears in several other studies (Chhabra and Jacobs, 2008; Todorov, 2009; Alessandro and Nori, 2012). Chhabra and Jacobs (2008) propose a method called Greedy additive regression (GAR). A library of task-specific actuations (synergies) are kept in memory. When a new task has to be performed, a suitable actuation is initially searched in the linear span of these synergies. If the lowest task-error is above a certain threshold, the task will be solved via traditional methods (e.g., feedback error learning), and the obtained actuation will be added to the library. If the library already contains the maximum number of synergies allowed, the least used one will be removed. The obtained results suggest that the synergies synthesized via GAR outperform primitives based on PCA if the dynamical system is non-linear (planar kinematic chain), and there is no statistical difference for linear systems. However, no theoretical explanation is provided.

In the same vein, Todorov (2009) proved that, for a certain class of stochastic optimal control problems, an appropriate change of variable in the Bellman equation allows to obtain the optimal control policy as a linear combination of some primitives. These primitives are, in turns, solutions to other optimal control problems. Such a method has recently been tested in the context of character animation (da Silva et al., 2009). It is important to clarify that this theory provides a theoretical grounding to the compositionality of optimal control laws, but like GAR it does not provide a method to compute such primitives. In fact, although new efficient methods have been proposed recently, solving an optimal control problem remains quite computationally intense, and it might be unfeasible for systems with a large number of dof.

Another mathematical framework, that has recently been developed in the context of character animations, is based on the optimal anechoic mixture decomposition model, mathematically equivalent to the time-varying synergy decomposition. Specifically, complex kinematic animations are obtained by mixing primitive source signals that are learned from motion captured data (Mezger et al., 2005; Park et al., 2008a,b; Giese et al., 2009). Within this framework a number of interesting results have been achieved, including a mathematical proof of stability properties for groups of characters that interact in various ways (Mukovskiy et al., 2011).

The procedure presented by Alessandro et al. (2012) is grounded on a method to solve generalized reaching tasks called dynamic response decomposition (DRD). In this context, a task is defined as a list of constraints on the values of the state variables at given points of time. Initially, a state-space solution is computed by interpolating these constraints by means of a set of dynamic responses (i.e., evolutions of the state variables); then, inverse dynamics is used to obtain the corresponding actuations. Based on this technique, the following two-phase procedure allows to synthesize a set of synergies. An extensive collection of generic actuations are used to generate the system dynamic responses (exploration phase); in a second stage (reduction phase), they are used to interpolate a small set of tasks. The corresponding actuations proved to be effective synergies for additional reaching tasks on a simulated planar kinematic chain. Like GAR, this procedure generates synergies in the form of feedforward controllers, and it allows to modify incrementally the library of synergies. However, DRD provides a computationally fast method to solve the task. This technique has proved its efficacy empirically, but a solid theoretical grounding is still lacking.

Most of the methods presented so far require an accurate analytical model of the system dynamics. Such a model is not always available, and for certain robots, it might be difficult to identify. Todorov and Ghahramani (2003) propose a method to synthesize synergies by means of unsupervised learning. Their work emphasizes the role of muscle synergies in an hypothetical hierarchical control scheme similar to the one proposed by Safavynia and Ting (2012): receptive fields translate sensory signals to internal variables, and muscle synergies translate high-level control signals applied to these variables to actual muscle contractions. From this perspective, receptive fields along with motor primitives must form an inverse model of the sensory-motor system. This mapping is learned by fitting a probabilistic model to a dataset of sensory-motor signals generated by actuating the robot with random pulses. The use of the learned synergies as low-level controllers substantially reduces the time needed to learn a desired policy, however, their capability to generalize to additional control laws is not explicitly tested.

Alessandro and Nori (2012) define synergies as parameterized functions of time that serve as feedforward controllers. The identification procedure consists in finding the values of the parameters such that appropriate linear combinations of the resulting synergies drive the dynamical system over a set of desired trajectories (training set). The synergies identified are then tested for generalization; the idea is to evaluate to which extent they can generate actuations that drive the system along a new group of trajectories (testing set). This procedure has been evaluated successfully in simulation and does not require the analytical form of the system dynamics. However, it is computationally very intense as it involves heavy optimizations. In essence, this work proposes a new formal definition of the concept of muscle synergies: elementary controls that are evaluated in terms of task-performance (i.e., tracking error), rather then in terms of approximation of the input-space.

Thomas and Barto (2012) formulate the problem of primitive (i.e., synergy) discovery within the framework of reinforcement learning. In this case, the problem that the agent has to solve is a Markov decision process (MDP), and each primitive is a parameterized feedback control policy. The idea is to identify the optimal parameters that maximize the expected reward for a given task, when the control is restricted to linear combinations of the learned primitives. This method is tested on a simulated planar kinematic chain actuated with artificial muscles. Primitives are identified on reaching tasks, and they are successfully tested in a scenario that involve reaching and avoiding obstacles. This work clearly shows the advantage of a synergy-based framework in terms of learning speed of novel control policies. This method is in essence similar to the one proposed by Alessandro et al. (2012), however, it identifies complete feedback control policies rather then single feedforward synergies.

The time-varying synergy model greatly reduces the dimensionality of the problems by encoding actuations with synergy-coefficients, however, at the same time it introduces a complication. As the new input variables are piecewise constant, it is difficult (although possible) to implement feedback loops. The synchronous model ameliorates this problem and, to some extent, it allows adapting traditional control strategies to the new reduced-dimensional control input.

Some researchers employ the synchronous synergy model to control the tendon-driven robotic ACT hand (Deshpande et al., 2013) in a reduced dimensional space (Rombokas et al., 2011; Zhang et al., 2011; Malhotra et al., 2012). Similarly to Todorov and Ghahramani (2003), dimensionality reduction is applied both in the sensory space and in the actuation space. The “observation synergies” transform sensory readings (tendon lengths) into a lower dimensional variable; the “control synergies” translates synergy-coefficients (as defined in section 2) to motor signals. Model adaptive control and PIDs are applied to the reduced dimensional input, and allow the robotic hand to perform tasks like writing (Rombokas et al., 2011; Malhotra et al., 2012) and playing piano (Zhang et al., 2011). The synergy matrices (observation and control) are computed by applying PCA and NMF to a dataset of tendon-lengths obtained as a result of defined hand motions. It is noteworthy that the more similar this motions are to the ones required to solve the task, the better the quality of the obtained synergy-based controller. This is clearly not surprising, but it highlights the importance of task-related variables in the formation of muscle synergies (Todorov et al., 2005).

Marques et al. (2012) identify synchronous synergies by means of an unsupervised Hebbian-like algorithm that captures the correlations between motor signals and sensory readings. Each synergy thus summarizes the levels of correlation between each motor and one of the sensors. The time modulation of each synergy to solve a given task is then obtained by means of a supervised learning procedure that aims at reducing the task error. Unlike many other works in robotics, the exploratory strategy proposed to generate the dataset of sensory-motor data does not exploit any prior information about the desired motor tasks, therefore muscle synergies are implicitly interpreted as patterns of motor coordinations that solely reflect the biomechanical constraints of the robot. This method has been tested on a single-joint tendon driven robot.

In the context of robotic hands, many researchers adopted the idea of postural-synergies, or eigengrasps. This concept derived by the observation that the variability of finger postures during human grasps can be explained by a few principal components (Santello et al., 1998), i.e., eigengrasps. Similarly, constraining the finger-joints positions of a robotic hand in such a way that the useful grasping postures can be obtained by superposing a small number of components, would result in a substantial simplification of the graphing problem. Ciocarlie and Allen (2009) derived a theoretical formulation of the problem of stable grasping in the low dimensional space of the postural-synergies; such a formulation is further improved by Gabiccini et al. (2011) for complain grasps. These studies are further analyzed and discussed by Bicchi et al. (2011), who presented them from the point of view of modeling the process of grasping and active touch. Finally, Brown and Asada (2007) proposed a direct mechanical implementation of the eigengrasps. In all these works, the quantitative details of the postural-synergies are taken from human experiments and adapted to the robot mechanical structure; the problem of finding a set of synergies that is optimized for a given robotic hand is left as future research.

Reduced dimensionality based on postural synergies is also explored by Hauser et al. (2011) for the task of balancing a humanoid robot. The authors propose a mathematical formulation, as well as a method to construct kinematic synergies (i.e., predefined balance between joint positions) that are directly linked to task variables (e.g., for balance control, the center of pressure). Additionally, the synergies are constructed in such a way that the mapping from synergy coefficients to task variables is linear (similar to the work proposed by Nori and Frezza (2005) but in kinematic space). This allows to use a simple proportional-integral-derivative controller (PID) on the synergy coefficients to control the center of pressure of the robot, as long as the movements are slow enough to neglect dynamic disturbances. The proposed method is demonstrated both in simulation and in a real humanoid.

As a final note, it is important to say that the concept of modularity has been employed in robot control in many other ways. In most of these works modules are defined as kinematic-based controllers that are combined sequentially to obtain complex joint trajectories (Khansari-Zadeh and Billard, 2011; Ijspeert et al., 2013). In this regard, these works are more related to the concept of kinematic stroke than to muscle synergies (Pollick et al., 2009). These works are out of the scope of this paper, as we focus on controllers that, in accordance with the models of muscle synergies, are based on (parallel) superpositions of primitives in input-space.

## 5. Conclusions and perspectives

The hypothesis of muscle synergies, that proposes a modular organization of the neural circuitry involved in muscle coordination, has been proved very difficult to validate or falsify (Tresch and Jarc, 2009). As discussed in section 3, a substantial body of evidence in favor of this hypothesis comes from the observation that the main components of EMG recordings are robust across behaviors, biomechanical contexts, and individuals. In addition, the successful control of artificial agents confirm the computational feasibility of the hypothesized synergy-based controller (section 4). However, there also exist experiments that, for the case of the human hand, seem to disprove the hypothesis of muscle synergies (Kutch et al., 2008; Valero-Cuevas et al., 2009). As a matter of fact, there is no real consensus yet on whether muscle synergies effectively represent a modular organization of the CNS, or they merely result from the methodology employed during the experiments.

The works that are based on the control of artificial agents (e.g., musculoskeletal models, robots, and animated characters) clarify the importance of evaluating synergies in task-space. In this context, the idea is to synthesize a set of synergies that guarantees the accomplishment of the desired tasks (Figure 3, red arrows). On the contrary, the main focus of experimental motor control has been to identify the synergies that better reconstruct the recorded EMG dataset (Figure 3, continuous green arrows), and to understand their neural substrate. This approach implicitly assumes that a well reconstructed input signal leads to the observed task performance. Given the non-linear dynamics of the musculoskeletal system, this assumption might not hold. For this reason, in our view the hypothesis of muscle synergies should be tested by validating an input–output model (i.e., from muscle activations to task-variables), rather than fitting a model of the input data alone (Figure 3, dashed green arrow). In fact, we could speculate that muscle synergies encode a form of body schema (Hoffmann et al., 2010) that allows translating intentions to motor plans (i.e., the inverse dynamic model of the musculoskeletal system) (Torres-Oviedo and Ting, 2010).

The concept of functional synergies represents a first attempt to relate muscle synergies to task variables. However, as discussed in section 4.2, EMG and task-level components are assumed to be activated by the same coefficients. This assumption cannot hold in general because the musculoskeletal system is non-linear; rather, input-space and task-space coefficients should be related by a non-linear mapping (as described by Alessandro et al., 2012). To address this issue, one should go beyond the use of NMF, and develop novel techniques that do not impose a linear mapping between the two sets of coefficients. Additionally one could try to reconstruct the task-variables with more general non-linear methods instead of imposing a linear combination also at the task level. In the same spirit of the procedure used so far, such a technique should optimize the reconstruction error of the EMG signals, and constrain a good fit of the task-variables. In any case, the generality of the extracted functional synergies should be tested. To the best of our knowledge, the model of functional synergies was never used as a predictive framework. It would be extremely interesting to evaluate the extent to which functional synergies identified during the execution of a certain set of tasks, are able to predict the muscle activations observed during the execution of another task that involve the same task variables. If such prediction was unsuccessful, the experimenter could conclude that the identified muscle synergies do not really encode the hypothesized biomechanical functionalities, or that the same functionalities might be encoded by different synergies. In general, the model of muscle synergies has very seldom been used to make predictions.

An alternative strategy to verify the relationship between muscle synergies and task execution (Figure 3, dashed green arrow), is to evaluate if they can account for task-related variations of single movement executions (Delis et al., 2013). In practice, one might assess the capability of these synergies to decode each repetition of different motor tasks. In other words, one should be able to classify the motor tasks from the activation coefficients of the extracted synergies. If the decoding capability is satisfactory, one might conclude that the synergies not only constitute a low dimensional, but also a functional representation of the motor commands. This idea might be used to develop novel extraction algorithms that include task decoding objectives directly in the optimization procedure. The identified synergies would then maximize not only the reconstruction of the original motor patterns, but also the capability of disambiguating task-relevant trial-to-trial variations. Unlike the dimensionality reduction methods used so far, this approach would rely on supervised learning techniques to exploit information about the task. Possible alternatives to standard extraction algorithms include energy constrained discriminant analysis (Philips et al., 2009), the discriminant NMF (Buciu and Pitas, 2004), and the hybrid discriminant analysis (Yu et al., 2007).

The use of single-trial analysis, like the decoding strategy proposed above, may be useful for addressing some open problems that are relevant to this review. First, the development of such techniques may be useful to identify muscle activation components of relatively low amplitude that reflect unique information about the task (Quiroga and Panzeri, 2009); such components would be completely lost if an average across several trials is performed prior to the analysis. Second, such single-trial analysis techniques may be used to investigate the existence of trial-to-trial correlations across synergy activations, and to evaluate their functional role in controlling and performing task-related movement (Golledge et al., 2003; Schneidman et al., 2003). Finally, approaches based on single-trial analysis of neural activity could also be instrumental in clarifying the existence of a neural basis for the muscle synergies (Hart and Giszter, 2004, 2010; Nazarpour et al., 2012; Ranganathan and Krishnan, 2012). For example, they could in principle be applied to decode the task from single-trial neural population patterns that regulate the activation of synergies, and also to determine which patterns encode task differences, and which carry additional or independent information to that carried by other patterns (Delis et al., 2010).

Finally, an important aspect that is worth discussing is the role of feedback loops. In the case of synchronous synergies, the time course of the mixing coefficients can be adjusted on-line by means of appropriate feedback controllers; this is the reason of the popularity of such a model in the context of robotics. On the contrary, the models of temporal and time-varying synergies, in which the actuation time course are directly embedded in the synergies themselves, naturally represent feedforward controllers. As a result, the evolution of the task-variables intimately depends on the initial condition of the dynamical system. Alternatively, these synergies might be defined as functions of both time and state-variables; such an approach would characterize temporal and time-varying synergies as generators of complete control policies (Nori and Frezza, 2005; Todorov, 2009; Thomas and Barto, 2012).

In conclusion, we believe that the evidence reviewed here provides support for the existence of muscle synergies. However, many issues are still unresolved. A deeper investigation of the relationship between synergies and task variables might help to address some of the open questions. In general, a closer coordination between experimental and computational research might lead to a more objective assessment of the muscle synergy hypothesis in task-space, and a better understanding of the modularity of the CNS.

### Conflict of interest statement

The authors declare that the research was conducted in the absence of any commercial or financial relationships that could be construed as a potential conflict of interest.

## Footnotes

^1^The term *synergy* has also been used in the context of another motor control hypothesis, the uncontrolled manifold hypothesis (UMH) (Latash, 2010; Latash et al., 2010). In that context, the term refers to “a neural organization of a set of elemental variables (e.g., muscle contractions) with the purpose to ensure certain stability properties of a performance variable produced by the whole set (e.g., desired joint configuration)” (Latash et al., 2008). These studies are out of the scope of this paper, however, we will discuss the concept of M-modes, that has been introduced in the UMH but it is very similar to the definition of synergies we adopt in this manuscript.

^2^In control theory, a system is said to be controllable if an external input can move the system from any initial state to any final state in a finite time interval.
